# Cost Effectiveness of ACEIs/ARBs versus Amlodipine Monotherapies: A Single-Center Retrospective Chart Review

**DOI:** 10.3390/healthcare9070798

**Published:** 2021-06-25

**Authors:** Yazed AlRuthia, Fahad Alotaibi, Amr Jamal, Ibrahim Sales, Monira Alwhaibi, Nawaf Alqahtani, Sina M. AlNajrany, Khalid Almalki, Abdulaziz Alsaigh, Wael Mansy

**Affiliations:** 1 Department of Clinical Pharmacy, College of Pharmacy, King Saud University, P.O. Box 2454, Riyadh 11451, Saudi Arabia; 438105986@student.ksu.edu.sa (F.A.); isales@ksu.edu.sa (I.S.); malwhaibi@ksu.edu.sa (M.A.); 437102936@student.ksu.edu.sa (N.A.); Salnajrany@moh.gov.sa (S.M.A.); 435101508@student.ksu.edu.sa (K.A.); 435101407@student.ksu.edu.sa (A.A.); wsayed@KSU.EDU.SA (W.M.); 2 Family and Community Medicine Department, College of Medicine, King Saud University, P.O. Box 3145, Riyadh 12372, Saudi Arabia

**Keywords:** hypertension, angiotensin-converting enzyme inhibitors, angiotensin receptor antagonists, calcium channel blockers, cost effectiveness, Saudi Arabia

## Abstract

The aim of this retrospective chart review study was to examine the cost effectiveness of angiotensin-converting enzyme inhibitors (ACEIs); angiotensin receptor blockers (ARBs); and dihydropyridine calcium channel blockers (CCBs) such as amlodipine, monotherapies in the management of essential hypertension among adult patients (≥18 years) without cancer, cardiovascular disease, and chronic kidney disease in the primary care clinics of a university-affiliated tertiary care hospital. Patients were followed up for at least 12 months from the initiation of therapy. Propensity score bin bootstrapping with 10,000 replications was conducted to generate the 95% confidence intervals (CI) for both treatment outcome (e.g., reduction of the systolic (SBP) and diastolic blood pressures (DBP) in mmHG) and the cost (e.g., costs of drugs, clinic visits, and labs in Saudi riyals (SAR)). Among the 153 included patients who met the inclusion criteria, 111 patients were on ACEIs/ARBs, while 44 patients were on amlodipine. On the basis of the bootstrap distribution, we found that the use of ACEIs/ARBs was associated with an incremental reduction of SBP of up to 4.46 mmHg but with an incremental cost of up to SAR 116.39 (USD 31.04), which results in an incremental cost effectiveness ratio (ICER) of SAR 26.09 (USD 6.95) per 1 mmHg reduction with 55.26% level of confidence. With regard to DBP, ACEIs/ARBs were associated with an incremental reduction of DBP of up to 5.35 mmHg and an incremental cost of up to SAR 144.96 (USD 38.66), which results in an ICER of SAR 27.09 (USD 7.23) per 1 mmHg reduction with 68.10% level of confidence. However, ACEIs/ARBs were less effective and costlier than amlodipine in reducing SBP and DBP with 44.74% and 31.89% levels of confidence, respectively. The findings of this study indicate that the use of ACEI or ARB as a monotherapy seems to be more effective than amlodipine monotherapy in the management of essential hypertension in primary care settings with minimal incremental cost.

## 1. Introduction

Hypertension (HTN) is one of the most prevalent chronic diseases worldwide. It results in serious consequences, such as retinopathy, neuropathy, nephropathy, stroke, and myocardial infarction if left untreated [1,2]. This is concerning since many patients would not notice that they have HTN unless they have one of the above-mentioned micro or macrovascular complications [3]. Therefore, early detection and proper management of HTN is necessary to stave off its serious complications. The management of HTN is multifactorial and involves lifestyle modifications such as exercise, reduced intake of salt, weight loss, and increased fiber intake, as well as prescription medications [3]. There are various antihypertensive medications that are commonly prescribed for the management of HTN [4]. The choice of antihypertensive medications depends on the patient characteristics and severity of HTN, which is defined as a persistent elevation of blood pressure above 140 mmHg for systolic blood pressure (SBP) and/or more than 90 mmHg for the diastolic blood pressure (DBP) [5]. Therefore, the Joint National Committee on prevention, detection, evaluation, and treatment of high blood pressure (JNC8) report has categorized HTN into different stages on the basis of the severity of HTN and patient medical characteristics [6]. The prehypertensive stage refers to those with SBP between 120 and 139 mmHg or DBP between 81 and 89 mmHg [6]. Essential or primary HTN, in which no identifiable cause is present, represents more than 90% of HTN cases [7]. Angiotensin-converting enzyme inhibitors (ACEIs), angiotensin receptor blockers (ARBs), diuretics (e.g., hydrochlorothiazide and furosemide), and dihydropyridine calcium channel blockers (CCBs) (e.g., amlodipine) are some examples of commonly utilized classes of antihypertensive medications in the management of the first stage of HTN [6]. However, if the HTN could not be controlled by one medication, another antihypertensive agent can be added in order to bring the blood pressure down to normal (<140/90 mmHg or <120/80 mmHg) [6]. Although the Systolic Blood Pressure Intervention Trial (SPRINT) has found that the intensive treatment of HTN (e.g., <120 mmHg) was associated with lower risk of major complications, such as myocardial infarction, stroke, heart failure, and death [8], other studies have found that no significant difference in the rates of major complications between the standard (e.g., < 140/90 mmHg) and the intensive treatment approaches (e.g., <120 mmHg) [9,10,11,12].

The use of antihypertensive drugs as monotherapy in the management of essential HTN is uncommon since different antihypertensive medications are mostly used in combination with other antihypertensive medications belonging to different classes or with diuretics [6]. However, several studies have compared the effectiveness of different antihypertensive monotherapies with conflicting results. In a systematic review and meta-analysis that included 208 clinical trials with 94,305 patients, most antihypertensive drugs were associated with 10 to 15 mmHg and 8 to 10 mmHg reductions in SBP and DBP, respectively, when used as a monotherapy [13]. ARBs have shown to be more cost-effective than CCBs, β-blockers, and ACEIs in most comparisons based on a systematic review conducted by Park et al. [14]. However, the rates of fatal heart disease, non-fatal myocardial infarction, and all-cause mortality were not significantly different between lisinopril and amlodipine based on the findings of Antihypertensive and Lipid-Lowering Treatment to Prevent Heart Attack Trial (ALLHAT) [15]. Moreover, diuretics and β-blockers were found to be more cost-effective in the management of HTN than ACEIs or CCBs based on the results of an observational population-based study in Brazil [16]. Intraclass differences among ARBs were reported on the basis of a comprehensive literature review that reviewed the safety, efficacy, and cost of irbesartan in the management of essential HTN; irbesartan was found to be the most preferred antihypertensive agent in comparison to other ARBs as well as other antihypertensive alternatives [17]. Nonetheless, amlodipine was associated with lower cost and better efficacy in preventing stroke and myocardial infarction in comparison to ARBs according to two Markov-based analyses [18,19]. However, other economic evaluations have shown ARBs to be more cost-effective in delaying the progression of nephropathy among different hypertensive patient populations with diabetes [20,21,22,23,24,25]. In contrast, amlodipine was found to be more cost-effective than ACEIs, such as enalapril, in the management of mild-to-moderate essential HTN using one-year patient-level data from a single clinical trial [26]. Thus, the evidence seems to be controversial about the effectiveness of ACEIs/ARBs versus amlodipine and other CCBs in the management of essential HTN.

In Saudi Arabia, the utilization rates of different antihypertensive medications are high due to the high prevalence of essential HTN among the Saudi population, which is believed to be over 50% among those aged 55 years and above [27]. ACEIs/ARBs and CCBs are commonly prescribed antihypertensive agents that are used as a monotherapy in the management of first two stages of HTN in Saudi Arabia [28]. However, the cost-effectiveness of different antihypertensive medications used in combination or alone has not been examined before in Saudi Arabia. Therefore, the aim of this study was to examine the cost-effectiveness of ACEIs/ARBs versus amlodipine in terms of SBP and DBP reductions when used as monotherapies in the management of essential hypertension using real-world data from Saudi Arabia.

## 2. Methods

### 2.1. Study Design and Setting

This was a single-center retrospective chart review study that was conducted at King Saud University Medical City in Riyadh, Saudi Arabia. Electronic health records of adult patients (≥18 years) visiting family practice clinics were reviewed to identify patients with essential HTN treated with a single antihypertensive medication for at least 12 months. Patients with cancer, cardiovascular disease, chronic kidney disease, and/or stroke, as well as those with secondary HTN (e.g., hyperthyroidism, kidney disease, adrenal disease) were excluded. Age, gender, comorbidities (e.g., diabetes, dyslipidemia), number of prescription medications, baseline SBP and DBP, duration of follow-up in months from the time where patients were diagnosed with HTN to the next annual clinic visit, follow-up SBP and DBP, and antihypertensive medications (e.g., names, dosages, frequency of administration) were reviewed and collected. Moreover, the utilization of different health services, such as labs and imaging studies, were collected. The electronic health record system also known as E-Sihi was implemented at King Saud University Medical City in mid-2015. Therefore, the investigators decided to recruit patients who were first diagnosed with essential HTN and started on amlodipine or ACEIs/ARBs (lisinopril, captopril, and irbesartan) anytime between July 2015 and June 2018 to ensure complete data collection with no missing observations since it is highly unlikely to find patients with complete data prior to the implementation of E-Sihi. The retrospective data collection started on 21 January 2018 and ended on 27 June 2019.

### 2.2. Data Analysis

Using Wilcoxon–Mann–Whitney test, allocation ratio of 1 (amlodipine) to 3 (ACEIs/ARBs), α = 0.05, β = 0.2, power of 0.8, effect size of d = 0.52, we estimated the minimum sample size to be 144 patients (36 patients on amlodipine and 108 patients on ACEIs/ARBs). The outcome was defined as the difference between the baseline and follow-up SBP and DBP in mmHG. On the other hand, the costs of prescription medications and healthcare services (e.g., clinic visits, labs, imaging studies) were retrieved from the Saudi Ministry of Health cost center. Chi-squared test, Fisher’s exact test, Student’s *t*-test, and one-way ANOVA were conducted as appropriate to compare the characteristics of patients on amlodipine and ACEIs/ARBs (lisinopril, captopril, and irbesartan). Propensity score matching was conducted on the basis of baseline SBP and DBP, age, gender, follow-up period, body mass index (BMI), and Charlson’s comorbidity index (CCI) score [29]. Bias-corrected and accelerated (BCa) non-parametric bootstrapping with 10,000 replications was conducted to generate the 95% confidence intervals (CI) for the cost in Saudi Riyals (SAR) and outcome (e.g., difference between the baseline and follow-up SBP and DBP). The incremental cost effectiveness ratio (ICER) was calculated on the basis of the following formula:

$$ICER=\frac{Mean\;cost\;in\;SAR\;for\;ACEIs\;or\;ARBs-Amlodipine}{Mean\;\;\;SBP\;or\;DBP\;reduction\;in\;mm\;Hg\;for\;ACEIs\;or\;ARBs-Amlodipine\;}$$

All statistical analyses were conducted using SAS^®^ version 9.4 (SAS^®^ institute Inc., Cary, NC, USA).

## 3. Results

### 3.1. Patient Characteristics

**Figure 1:**
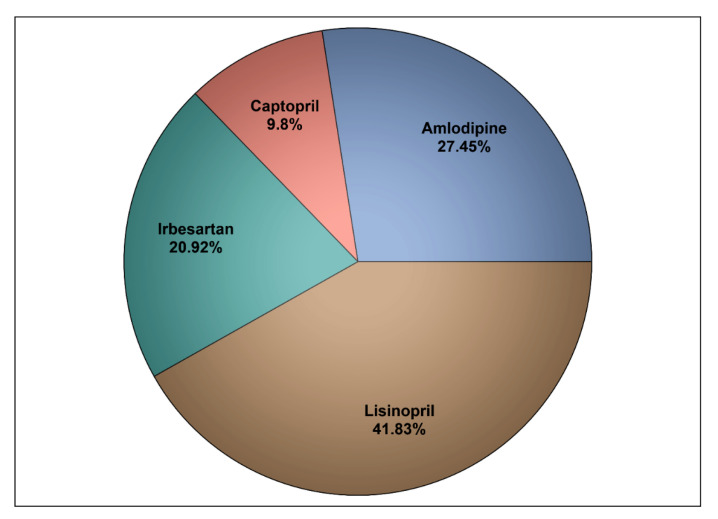
The utilization rates of ACEIs/ARBs and amlodipine among the study sample.

Out of the 2000 medical records that were reviewed for patients who have been diagnosed with HTN, 153 patients met the inclusion criteria and were included in the analysis. A total of 42 patients were taking amlodipine (27.45%) (*n* = 42), and 111 patients (72.55%) were taking ACEIs or ARBs (e.g., irbesartan, captopril, and lisinopril), as shown in Figure 1. Patients’ mean age was 56 years, their mean body mass index (BMI) was 31, they were followed up for a mean duration of 13.84 months, and most of them were female (56.21%). Those on amlodipine had significantly lower mean number of prescription medications and CCI score in comparison to their counterparts on ACEIs/ARBs (*p* < 0.05), as shown in Table 1.

### 3.2. The Costs and Outcomes of ACEIs/ARBs versus Amlodipine for HTN Management

**Figure 2:**
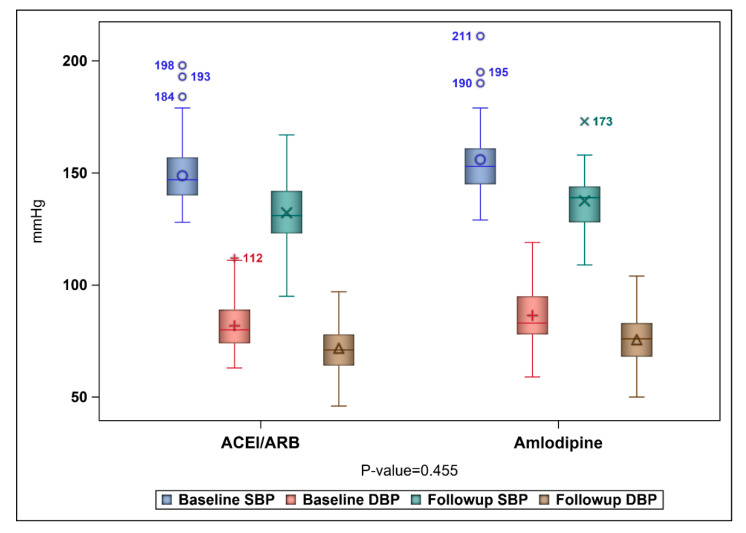
Box plot of baseline and follow-up SBP and DBP for patients on ACEIs/ARBs and amlodipine. The box represents the 25th and 75th percentiles, and the whiskers are the upper and lower adjacent values. The following shapes, 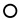, 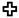, 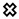 and 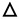 represent the means of the baseline SBP, baseline DBP, follow-up SBP, and follow-up DBP, respectively. The outside written values are outliers.

**Figure 3:**
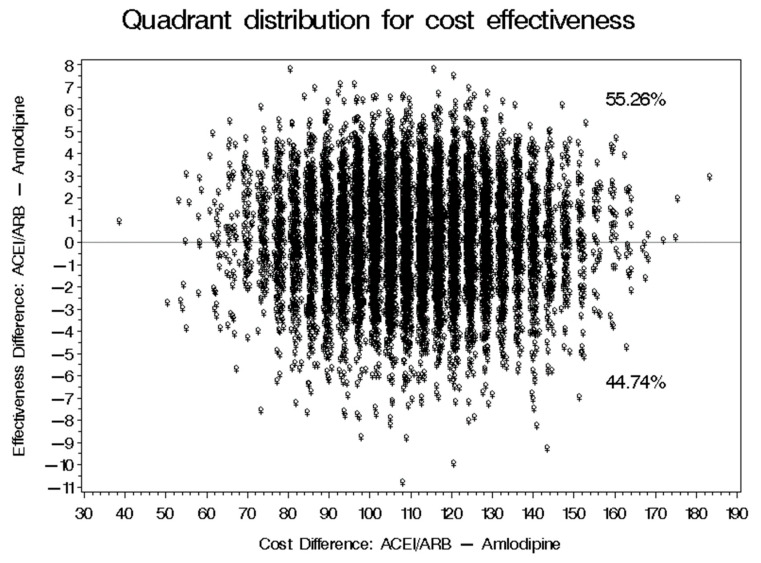
The probabilistic sensitivity analysis of the cost effectiveness of ACEIs/ARBs versus amoldipine in the management of high SBP.

**Figure 4:**
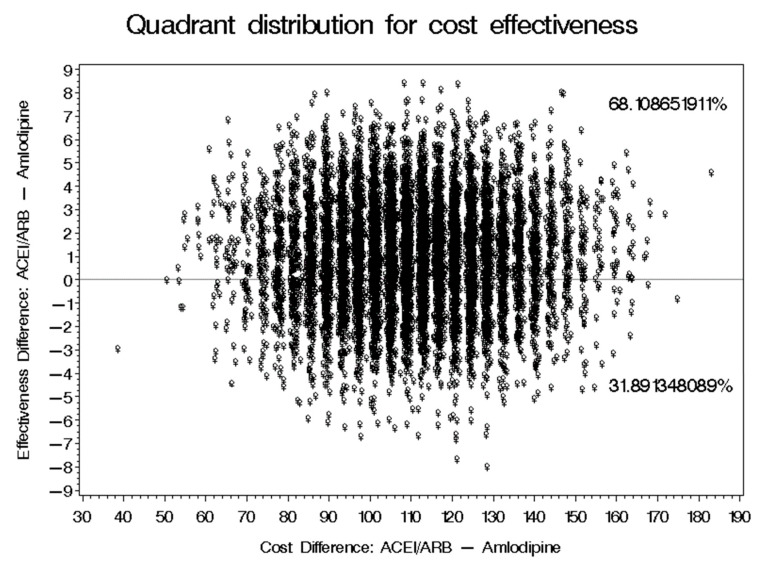
The probabilistic sensitivity analysis of the cost effectiveness of ACEIs/ARBs versus amoldipine in the management of high DBP.

No significant difference in the baseline and follow-up SBP and DBP for patients on ACEIs/ARBs and those on amlodipine was found as shown in Figure 2. The mean reductions of SBP for patients on ACEIs/ARBs and amlodipine were 16.54 ± 12.42 mmHg and 18.43 ± 17.31 mmHG, respectively. On the other hand, the mean reductions of DBP for patients on ACEIs/ARBs and amlodipine were 10.04 ± 12.16 mmHg and 10.83 ± 14.10 mmHG, respectively. The mean costs for patients on ACEIs/ARBs and amlodipine were SAR 1193.60 and SAR 1097.50, respectively, as shown in Table 2. The ICER of ACEIs/ARBs versus amlodipine for SBP was SAR-50.89 per 1 mmHg reduction, which means that the use of amlodipine was associated with a saving of SAR 50.89 for each incremental 1 mmHG reduction in SBP. However, 95% CIs BCa for the difference in cost and SBP reduction were [SAR 53.12–SAR 116.39] and [−4.53 mmHg–4.46 mmHg], which translates into an ICER for the use of ACEIs/ARBs versus amlodipine that would range between SAR-11.73 and SAR 26.09 per 1 mmHg reduction. On the basis of the bootstrap distribution, the use of ACEIs/ARBs would result in a greater reduction of SBP that can be as large as 4.46 mmHG and higher cost that can be as high as SAR 116.39 (USD 31.04) with 55.26% confidence level in comparison to amlodipine. However, ACEIs/ARBs can result in less reduction of SBP that can be 4.53 mmHg lower than amlodipine with higher cost that can be as high as SAR 116.39 (USD 31.04), as mentioned earlier with 44.74% level of confidence, as shown in Figure 3. On the other hand, the ICER of ACEIs/ARBs versus amlodipine for DBP was SAR-120.53 per 1 mmHg, which means that the use of amlodipine was associated with a saving of SAR 120.53 for each incremental 1 mmHG reduction in DBP. However, BCa 95% CIs for the difference in cost and DBP reduction were [SAR 76.72–SAR 144.96] and [−3.35 mmHg–5.35 mmHg], which translates into an ICER that would range between SAR 22.9 and SAR 27.09 per 1 mmHg reduction. This means that ACEIs/ARBs would result in a greater reduction of DBP that can be as large as 5.35 mmHg and higher cost that can be as high as SAR 144.96 (USD 38.66) with 68.11% confidence level in comparison to amlodipine. However, ACEIs/ARBs can result in lower reduction of DBP that can be 3.35 mmHg lower than amlodipine, with higher cost that can be as high as SAR 144.96 (USD 38.66) with 31.89% confidence level, as shown in Figure 4.

## 4. Discussion

The use of monotherapy in the management of HTN is uncommon [6]. However, understanding the impact of different antihypertensive medications when used as a monotherapy using real-world data is important in designing effective treatment plan for essential HTN, especially in a country with high incidence and prevalence rates of HTN such as Saudi Arabia [27]. ACEIs/ARBs and amlodipine are commonly prescribed for the management of the first two stages of essential HTN in Saudi Arabia [28]. These antihypertensive medications are mostly used in combination with other medications belonging to different antihypertensive classes [6]. Therefore, identifying patients with essential HTN managed with a single antihypertensive medication, which is the case in this study, was not an easy task. Although several studies have compared the efficacy and cost effectiveness of different ACEIs/ARBs and amlodipine in the management of essential HTN, the findings of these studies should be carefully interpreted due to the substantial variability of the patient characteristics and the acquisition costs of antihypertensive drugs. Moreover, these studies were not conducted among Middle Eastern or Saudi patient populations [13,14,16,17,18,19]. Therefore, examining the impact of ACEIs/ARBs and amlodipine on SBP and DBP when used as a monotherapy among patients with essential HTN in Saudi Arabia would be very informative to health practitioners and policy makers.

In this study, the use of ACEIs/ARBs were associated with larger reduction in both SBP and DBP than amoldipine in most of the 10,000 replications in the bootstrapping accounting for patients’ age, gender, CCI score, follow-up period, and BMI through the propensity score matching. However, this comes at an incremental cost that ranges between SAR 53.12 (USD 14.16) and SAR 144.96 (USD 38.66) per 1 mmHg reduction in SBP or DBP. These findings are consistent with the previously published studies which found that ACEIs/ARBs, such as lisinopril and irbesartan, were more cost-effective than amlodipine in the management of essential HTN [14,17]. These incremental benefits were more evident when ACEIs/ARBs, such as valsartan, were compared to amlodipine among patients with type 2 diabetes and microalbuminuria [25]. This is very important given the high prevalence and incidence rates of diagnosed and hidden diabetes in Saudi Arabia especially among patients with HTN [27,30]. However, the mean reductions in both SBP and DBP among patients on amlodipine were relatively larger than the ones seen with ACEIs/ARBs. Moreover, amlodipine has shown to lead to better reductions and lower cost in comparison to ACEIs/ARBs in 44.74% and 31.89% of the 10,000 bootstrapped replications for SBP and DBP, respectively. Additionally, in two state transition (Markov) models that compared the 5-year costs and quality-adjusted life-years (QALYs) of amlodipine versus ACEIs/ARBs using utility data and costs of myocardial infarction and stroke in China and Taiwan, amlodipine was found to be dominant since it was associated with incremental QALYs and lower costs [18,19]. However, these findings are based on two transition models that used utilities and costs from two Far Eastern countries with significantly lower incidence and prevalence rates of diabetes in comparison to Saudi Arabia where the prevalence of HTN among diabetic patients can be as high as 61% [30,31]. In addition, ACEIs/ARBs are still believed to lead to better outcomes in the management of HTN according to most published research [20,21,22,23,24].

Although this is the first study, to our knowledge, to compare the cost effectiveness of ACEI/ARB to CCB monotherapy using real-world data in Saudi Arabia, several limitations must be acknowledged. First, this is a single-centered study with a relatively small sample size, which limits the generalizability of its findings. However, the study was conducted in a well-known public tertiary care hospital in the Saudi capital where patients from all segments of the Saudi population are cared for. Secondly, information bias cannot be ruled out as the data were retrieved from the electronic healthcare records during a specified timeframe (i.e., July 2015 to June 2018), which also increases the risk of selection bias. Nevertheless, obtaining real-world data from electronic healthcare records in Saudi Arabia where multiple barriers to access to healthcare data suitable for conducting economic evaluation exist is a daunting task [32]. Moreover, the study examined the cost effectiveness of ACEIs/ARBs as a class of antihypertensive medications versus amlodipine rather than comparing individual ACEIs/ARBs versus one another and amlodipine. Although it is largely believed that ACEIs and ARBs are generally equally effective [33], some studies have shown that some ARBs, such as irbesartan, are more effective than other ACEIs/ARBs [20,21,22,23,24].

## 5. Conclusions

Effective management of HTN is essential in preventing major complications, such as stroke and myocardial infarction. Therefore, early detection and treatment of HTN using the most effective and affordable antihypertensive medication is important. In this study, ACEIs/ARBs were shown to be more effective with reasonable incremental cost in managing essential HTN in comparison to amlodipine. Future studies should examine the cost effectiveness of different monotherapies as well as combination therapies in the management of HTN using larger sample sizes and more robust study designs.

## Figures and Tables

**Table 1:** Patients’ baseline characteristics.

Characteristic	ACEIs/ARBs (*n* = 111)	Amlodipine (*n* = 42)	*p*-Value	Total
Gender				
Male	51 (45.95)	16 (38.10)	0.382	67 (43.79)
Female	60 (54.05)	26 (61.90)		86 (56.21)
Age	56.27 ± 11.61	55.76 ± 12.44	0.818	56.13 ± 11.80
Body mass index (BMI)	31.71 ± 5.97	30.36 ± 6.73	0.230	31.34 ± 6.19
Number of prescription medications	6.80 ± 3.33	4.62 ± 2.61	<0.001	6.20 ± 3.29
Charlson Comorbidity Index (CCI)	2 ± 1.16	1.47 ± 1.17	0.015	1.85 ± 1.18
Duration of follow-up in months	13.89 ± 1.89	13.71 ± 1.94	0.608	13.84 ± 1.90

**Table 2:** The mean reductions in blood pressure (SBP and DBP) and costs of ACEIs/ARBs versus amlodipine.

Variable	ACEIs/ARBs	Amlodipine	Mean Difference (95% CI)
Cost of treatment (SAR), mean ± SD	1193.60 ± 195.29	1097.50 ± 53.21	96.1 (53.12–116.39)
Mean reduction in SBP (mmHG), mean ± SD	16.54 ± 12.42	18.43 ± 17.31	−1.89 (−4.53–4.46)
Mean reduction in DBP (mmHG), mean ± SD	10.04 ± 12.16	10.83 ± 14.10	−0.79 (−3.35–5.35)

## Data Availability

The data are available upon reasonable request from the corresponding authors.

## References

[1] Lawes C.M., Hoorn S.V., Rodgers A. (2008). Global burden of blood-pressure-related disease, 2001. Lancet.

[2] Gaziano T., Bitton A., Anand S., Weinstein M.C. (2009). The global cost of nonoptimal blood pressure. J. Hypertens..

[3] Mensah G.A. (2019). Commentary: Hypertension Phenotypes: The Many Faces of a Silent Killer. Ethn. Dis..

[4] Wright J.M., Musini V., Gill R. (2018). First-line drugs for hypertension. Cochrane Database Syst. Rev..

[5] Unger T., Borghi C., Charchar F., Khan N.A., Poulter N.R., Prabhakaran D., Ramirez A., Schlaich M., Stergiou G.S., Tomaszewski M. (2020). 2020 International Society of Hypertension Global Hypertension Practice Guidelines. Hypertension.

[6] James P.A., Oparil S., Carter B.L., Cushman W.C., Dennison-Himmelfarb C., Handler J., Lackland D.T., LeFevre M.L., MacKenzie T.D., Ogedegbe O. (2014). 2014 evidence-based guideline for the management of high blood pressure in adults: Report from the panel members appointed to the Eighth Joint National Committee (JNC 8). JAMA.

[7] Poulter N.R., Prabhakaran D., Caulfield M. (2015). Hypertension. Lancet.

[8] Johnson K.C., Whelton P.K., Cushman W.C., Cutler J.A., Evans G.W., Snyder J.K., Ambrosius W.T., Beddhu S., Cheung A.K., Fine L.J. (2018). Blood Pressure Measurement in SPRINT (Systolic Blood Pressure Intervention Trial). Hypertension.

[9] Papademetriou V., Zaheer M., Doumas M., Lovato L., Applegate W.B., Tsioufis C., Mottle A., Punthakee Z., Cushman W.C., ACCORD Study Group (2016). Cardiovascular Outcomes in Action to Control Cardiovascular Risk in Diabetes: Impact of Blood Pressure Level and Presence of Kidney Disease. Am. J. Nephrol..

[10] Ruggenenti P., Perna A., Loriga G., Ganeva M., Ene-Iordache B., Turturro M., Lesti M., Perticucci E., Chakarski I.N., Leonardis D. (2005). Blood-pressure control for renoprotection in patients with non-diabetic chronic renal disease (REIN-2): Multicentre, randomised controlled trial. Lancet.

[11] Juraschek S.P., Appel L.J., Miller E.R., Mukamal K.J., Lipsitz L.A. (2018). Hypertension Treatment Effects on Orthostatic Hypotension and Its Relationship with Cardiovascular Disease. Hypertension.

[12] Mancia G., De Backer G., Dominiczak A., Cifkova R., Fagard R., Germano G., Grassi G., Heagerty A., Kjeldsen S.E., Laurent S. (2006). 2007 Guidelines for the management of arterial hypertension: The Task Force for the Management of Arterial Hypertension of the European Society of Hypertension (ESH) and of the European Society of Cardiology (ESC). Eur. Heart J..

[13] Paz M.A., De-La-Sierra A., Sáez M., Barceló M.A., Rodríguez J.J., Castro S., Lagarón C., Garrido J.M., Vera P., Coll-De-Tuero G. (2016). Treatment efficacy of anti-hypertensive drugs in monotherapy or combination. Medicine.

[14] Park C., Wang G., Durthaler J.M., Fang J. (2017). Cost-effectiveness Analyses of Antihypertensive Medicines: A Systematic Review. Am. J. Prev. Med..

[15] Jr J.T.W., Probstfield J.L., Cushman W.C., Pressel S.L., Cutler J.A., Davis B.R., Einhorn P.T., Rahman M., Whelton P.K., Ford C.E. (2009). ALLHAT Findings Revisited in the Context of Subsequent Analyses, Other Trials, and Meta-analyses. Arch. Intern. Med..

[16] Moreira G.C., Cipullo J.P., Martin J.F.V., Ciorlia L.A., Godoy M.R., Cesarino C.B., Cordeiro J., Lupino P.L., Ciorlia G., Burdmann E. (2009). Evaluation of the awareness, control and cost-effectiveness of hypertension treatment in a Brazilian city: Populational study. J. Hypertens..

[17] Maniadakis N., Gialama F. (2013). Comprehensive overview: Efficacy, tolerability, and cost-effectiveness of irbesartan. Vasc. Health Risk Manag..

[18] Chan L., Chen C.H., Hwang J.J., Yeh S.J., Shyu K.G., Lin R.T., Li Y.H., Liu L.Z., Li J.Z., Shau W.Y. (2016). Cost-effectiveness of amlodipine compared with valsartan in preventing stroke and myocardial infarction among hypertensive patients in Taiwan. Int. J. Gen. Med..

[19] Wu Y., Zhou Q., Xuan J., Li M., Zelt S., Huang Y., Yin H., Huang M. (2013). A Cost-Effectiveness Analysis between Amlodipine and Angiotensin II Receptor Blockers in Stroke and Myocardial Infarction Prevention among Hypertension Patients in China. Value Health Reg. Issues.

[20] Annemans L., Demarteau N., Hu S., Lee T.-J., Morad Z., Supaporn T., Yang W.-C., Palmer A.J. (2008). An Asian Regional Analysis of Cost-Effectiveness of Early Irbesartan Treatment versus Conventional Antihypertensive, Late Amlodipine, and Late Irbesartan Treatments in Patients with Type 2 Diabetes, Hypertension, and Nephropathy. Value Health.

[21] Li R., Zhang P., Barker L.E., Chowdhury F.M., Zhang X. (2010). Cost-Effectiveness of Interventions to Prevent and Control Diabetes Mellitus: A Systematic Review. Diabetes Care.

[22] Huang Y., Zhou Q., Haaijer-Ruskamp F.M., Postma M.J. (2014). Economic evaluations of angiotensin-converting enzyme inhibitors and angiotensin II receptor blockers in type 2 diabetic nephropathy: A systematic review. BMC Nephrol..

[23] Rodby R.A., Chiou C.F., Borenstein J., Smitten A., Sengupta N., Palmer A.J., Roze S., Annemans L., Simon T.A., Chen R.S. (2003). The cost-effectiveness of irbesartan in the treat-ment of hypertensive patients with type 2 diabetic nephropathy. Clin. Ther..

[24] Esposti L.D. (2010). Antihypertensive therapy among newly treated patients: An analysis of adherence and cost of treatment over years. Clin. Outcomes Res..

[25] Smith D.G., Nguyen A.B., Peak C.N., Frech F.H. (2004). Markov modeling analysis of health and economic outcomes of therapy with valsartan versus amlodipine in patients with type 2 diabetes and microalbuminuria. J. Manag. Care Pharm..

[26] Doyle J., Omvik P., Arikian S., Casciano J., Casciano R., Gonzalez M., Arocho R. (2001). A retrospective analysis comparing the costs and cost effectiveness of amlodipine and enalapril in the treatment of hypertension. Manag. Care Interface.

[27] El Bcheraoui C., Memish Z.A., Tuffaha M., Daoud F., Robinson M., Jaber S., Mikhitarian S., Saeedi M.A., AlMazroa M.A., Mokdad A.H. (2014). Hypertension and Its Associated Risk Factors in the Kingdom of Saudi Arabia, 2013: A National Survey. Int. J. Hypertens..

[28] Ali M.D. (2020). Cost analysis and utilization of antihypertensive drug therapy in Saudi Arabia. J. Pharm. Health Serv. Res..

[29] Molto A., Dougados M. (2014). Comorbidity indices. Clin. Exp. Rheumatol..

[30] Abdulghani H.M., AlRajeh A.S., AlSalman B.H., AlTurki L.S., AlNajashi N.S., Irshad M., Alharbi K.H., AlBalawi Y.E., AlSuliman Y., Ahmad T. (2018). Prevalence of diabetic comorbidities and knowledge and practices of foot care among diabetic patients: A cross-sectional study. Diabetes Metab. Syndr. Obes..

[31] Li Y., Teng D., Shi X., Qin G., Qin Y., Quan H., Shi B., Sun H., Ba J., Chen B. (2020). Prevalence of diabetes recorded in mainland China using 2018 diagnostic criteria from the American Diabetes Association: National cross sectional study. BMJ.

[32] AlRuthia Y., Alrashed S.A., Balkhi B., Aljamal M.S., Alsifri S., Alrumaih A.M., Alsabaan F., Alsaqa’Aby M., Al-Abdulkarim H.A., Altowaijri A.I. (2021). COVID-19 and Saudi Arabia public financing of prescription drugs: An opportunity for reform. Health Policy Technol..

[33] Tazkarji B., Ganeshamoorthy A., Auten B. (2015). Angiotensin-Converting Enzyme Inhibitors vs. Angiotensin Receptor Blockers. Am. Fam. Physician.

